# Microalgae Bioactives for Functional Food Innovation and Health Promotion

**DOI:** 10.3390/foods14122122

**Published:** 2025-06-17

**Authors:** José L. Guil-Guerrero, José A. M. Prates

**Affiliations:** 1 Departamento de Tecnología de Alimentos, Universidad de Almería, 04120 Almería, Spain; jlguil@ual.es; 2 CIISA—Centro de Investigação Interdisciplinar em Sanidade Animal, Faculdade de Medicina Veterinária, Universidade de Lisboa, Av. da Universidade Técnica, 1300-477 Lisboa, Portugal; 3 Associate Laboratory for Animal and Veterinary Sciences (AL4AnimalS), Av. da Universidade Técnica, 1300-477 Lisboa, Portugal

**Keywords:** bioactive compounds, chronic diseases, functional foods, microalgae composition, sustainable nutrition

## Abstract

Microalgae are increasingly recognised as sustainable, nutrient-dense sources of bioactive compounds with broad health-promoting potential. Rich in carotenoids, phenolics, polyunsaturated fatty acids, phycobiliproteins, sterols, and essential vitamins, microalgae offer a promising foundation for functional foods targeting chronic disease prevention. This narrative review explores the nutritional profiles and biological effects of key species, including Spirulina (*Limnospira platensis*), *Chlorella*, *Haematococcus*, and *Nannochloropsis*. Scientific evidence supports their antioxidant, anti-inflammatory, immunomodulatory, antimicrobial, and metabolic regulatory activities, contributing to reduced risks of cardiovascular, metabolic, inflammatory, and neurodegenerative disorders. Special emphasis is placed on the synergistic benefits of consuming whole biomass compared to isolated compounds and the technological strategies, such as encapsulation, cell wall disruption, and nutrient optimisation, that enhance the bioavailability of microalgal bioactives. Furthermore, the environmental advantages of microalgae cultivation, such as minimal land and freshwater requirements, carbon sequestration, and wastewater bioremediation, highlight their role in the transition toward sustainable food systems. Despite challenges related to high production costs, sensory attributes, scalability, and regulatory approval, advances in biotechnology, processing, and formulation are paving the way for their broader application. Overall, microalgae represent next-generation bioactive sources that promote human health and environmental sustainability, positioning them as key players in future functional foods and nutraceuticals.

## 1. Introduction

In recent decades, the global scientific and public health communities have increasingly recognised the pivotal role of bioactive compounds in supporting health and preventing disease. These compounds, while not classified as essential nutrients, exert significant physiological effects and are found naturally in various plant and marine foods. Common classes include phenolics, carotenoids, phytosterols, flavonoids, and polyunsaturated fatty acids (PUFAs), each associated with mechanisms that modulate oxidative stress, inflammation, immune response, and metabolic regulation [1]. The growing body of evidence linking the regular intake of bioactives to the reduction in risk factors for chronic non-communicable diseases, such as cardiovascular disease, type 2 diabetes, metabolic syndrome, cancer, and neurodegenerative disorders, has led to a surge in interest around functional foods, which are designed to deliver health benefits beyond basic nutrition [2,3].

Although traditional terrestrial plants remain important sources of bioactives, emerging attention is now turning to aquatic systems, especially microalgae, as next-generation functional food ingredients. Microalgae are photosynthetic, unicellular organisms capable of thriving in marine and freshwater environments. They represent a diverse group of over 30,000 known species, although only a handful are currently approved for human consumption [4]. Unlike conventional crops, microalgae can be cultivated under controlled conditions in bioreactors or open ponds, using minimal land, freshwater, and chemical inputs, making them a promising solution for sustainable food production in the face of environmental degradation, population growth, and climate change [5,6]. Microalgae can yield up to 20 times more protein per hectare than terrestrial crops such as soybeans while requiring approximately 90% less freshwater per kg of protein produced. Their year-round productivity and ability to grow on non-arable land using brackish or wastewater further enhance their environmental efficiency and sustainability profile [7].

Edible microalgal species such as Spirulina (recently reclassified as *Limnospira platensis*), *Chlorella vulgaris*, *Haematococcus pluvialis*, *Nannochloropsis gaditana*, and *Tetraselmis suecica* are particularly rich in nutritionally valuable and bioactive compounds. These include high-quality proteins; essential amino acids; omega-3 long-chain PUFAs, such as eicosapentaenoic acid (EPA, 20:5*n*-3) and docosahexaenoic acid (DHA, 22:6*n*-3); carotenoids (e.g., astaxanthin, β-carotene, and lutein); vitamins (A, B12, C, E, and K); phycobiliproteins; chlorophylls; polysaccharides; and minerals like iron, calcium, magnesium, and selenium [8,9,10]. Many of these nutrients are present in higher concentrations or more bioavailable forms than in terrestrial vegetables and grains, making microalgae especially appealing for use in vegan, vegetarian, and malnutrition-sensitive populations. These species were selected due to their dominant presence in the scientific literature, wide commercial availability, established safety profiles (e.g., GRAS status), and rich diversity of bioactive compounds, collectively representing the majority of the global microalgae market share in the food and nutraceutical sectors [4].

For instance, *Chlorella* and Spirulina species are renowned for their complete protein content, providing all essential amino acids in ratios comparable to high-quality animal proteins like eggs and milk [11,12]. *H. pluvialis* is the richest known natural source of astaxanthin, a potent antioxidant that protects cells from UV-induced and oxidative damage [13]. *Nannochloropsis* species excel in producing EPA, a long-chain omega-3 PUFA associated with cognitive support and lipid metabolism regulation [14,15]. Meanwhile, *Tetraselmis* spp. offer marine-derived phytosterols and carotenoids that have demonstrated benefits for cardiovascular health [16,17].

Beyond their exceptional nutritional value, microalgae offer significant environmental benefits that make them uniquely suited for sustainable food systems. These organisms exhibit rapid growth rates; some species can double their biomass within 24 h and can be cultivated on non-arable land using saline, brackish, or even wastewater, thereby avoiding direct competition with conventional agriculture [18]. Their high photosynthetic efficiency and remarkable capacity for carbon dioxide fixation position microalgae as a powerful tool for climate change mitigation; some species are capable of capturing over 1.8 kg of CO_2_ per kilogram of dry biomass produced [19]. These attributes are especially critical in the face of growing challenges such as land degradation, freshwater scarcity, and rising global temperatures, reinforcing the case for integrating microalgae into resilient food and energy systems.

From a health perspective, microalgal bioactives exert beneficial effects through multiple synergistic mechanisms. Carotenoids such as astaxanthin and β-carotene help neutralise reactive oxygen species (ROS), thereby mitigating oxidative stress and reducing cellular damage associated with ageing and chronic inflammation [20]. Phycocyanin, a pigment–protein complex derived from Spirulina, demonstrates potent antioxidant and neuroprotective properties by upregulating endogenous antioxidant enzymes and downregulating key inflammatory mediators [21]. Omega-3 PUFAs, mainly those from *Nannochloropsis* and *Schizochytrium*, have been shown to improve lipid profiles, enhance insulin sensitivity, and regulate inflammation via the peroxisome proliferator-activated receptor (PPAR) and AMP-activated protein kinase (AMPK) signalling pathways [22]. Additionally, sulphated polysaccharides from species such as *Porphyridium* and *Chlorella* support mucosal immunity and foster beneficial gut microbiota by acting as prebiotics [23].

**Figure 1:**
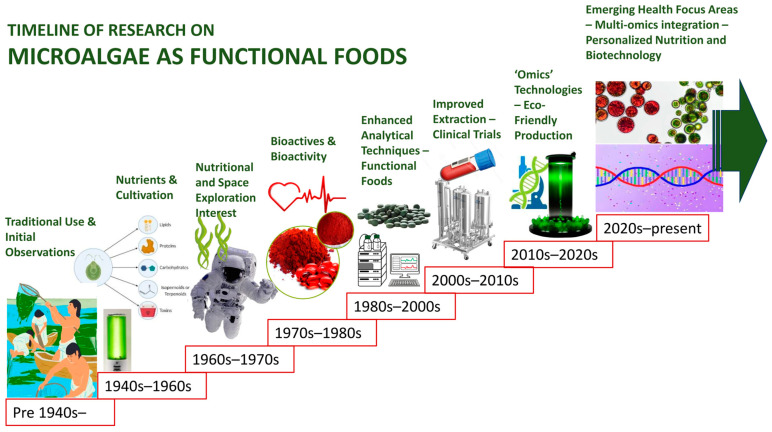
Timeline of research on microalgae as functional foods.

Despite their considerable potential, microalgae still face barriers to widespread adoption. Key challenges include high production and processing costs, batch-to-batch variability, difficulties in the extraction and standardisation of bioactive compounds, and limited consumer familiarity. Additionally, unfavourable sensory attributes, such as a fishy taste, dark green colour, and marine odour, can hinder acceptance in mainstream food markets [24,25]. However, ongoing advances in algal biotechnology, including improved strain selection, cell wall disruption techniques, microencapsulation, and food-grade emulsification, are increasingly addressing these limitations. Such innovations are enhancing the stability, sensory qualities, and bioavailability of microalgae-based ingredients in functional food products [26,27]. The trajectory of microalgae in food research and application has evolved markedly over recent decades. Scientific breakthroughs, regulatory approvals, and commercial product developments are collectively driving the integration of microalgae into mainstream nutrition [28,29]. A timeline of these pivotal milestones is illustrated in Figure 1, charting the progressive recognition of microalgae as functional food resources.

In light of growing scientific interest, this narrative review aims to comprehensively evaluate the health benefits and functional food potential of bioactive compounds derived from edible microalgae. It specifically addresses (i) the nutritional composition and biochemical properties of key species such as Spirulina, *Chlorella*, *Haematococcus*, and *Nannochloropsis*; (ii) their physiological roles in the prevention of chronic diseases; (iii) supporting evidence from in vitro, in vivo, and clinical studies; (iv) current applications in functional food and nutraceutical formulations; and (v) their environmental sustainability and role in shaping innovative food systems. By synthesising the current research and highlighting ongoing challenges, this review contributes to the advancing discourse on microalgae as a viable, health-promoting, and sustainable food resource for the future. This review is organised as follows: Section 2 outlines the methodology used for literature selection and analysis. Section 3 presents the main bioactive compounds derived from microalgae, including proteins, pigments, lipids, polysaccharides, and polyphenols. Section 4 discusses the health-promoting properties of these bioactives. Section 5 reviews evidence from preclinical and clinical studies that supports their functional benefits. Section 6 explores the application of microalgae in functional foods and nutraceuticals. Section 7 addresses the sustainability and innovation potential of microalgae in modern food systems. Finally, Section 8 concludes the review and highlights future research directions.

## 2. Materials and Methods

This narrative review was conducted using a structured, multi-phase narrative approach to comprehensively explore the health benefits and functional food applications of bioactive compounds derived from microalgae. The process began with a literature search designed to find current and high-quality scientific evidence. To gather relevant studies, three major scientific databases, PubMed, Scopus, and Web of Science, were searched systematically, with the final search completed in April 2025. The review focused on literature published in English and limited its scope to peer-reviewed journal articles, systematic reviews, clinical trials, and relevant book chapters. To maintain scientific rigour and relevance, grey literature, preprints, and conference abstracts were excluded.

The search strategy combined both free-text keywords and controlled vocabulary, connected by Boolean operators to ensure comprehensive results. Search terms included phrases such as “microalgae bioactives”, “microalgae functional foods”, “microalgae and chronic disease prevention”, “omega-3 from microalgae”, “carotenoids microalgae”, “phycobiliproteins AND microalgae”, “Spirulina AND human health”, “*Chlorella* bioactive compounds”, and “sustainable nutrition AND microalgae”.

The review prioritised publications from the last decade (2015–2025) to ensure coverage of recent advancements. However, earlier seminal works were also included to provide essential historical context. To ensure the review’s relevance to human health and nutrition, only studies that examined edible microalgal species such as Spirulina, *Chlorella*, *Haematococcus*, *Nannochloropsis*, and *Tetraselmis* were included. Special emphasis was placed on research exploring the nutritional composition, bioactive content, physiological effects, and applications of these microalgae in functional foods and nutraceuticals. Studies focusing exclusively on non-food applications, such as biofuel production or wastewater remediation, were excluded unless they presented incidental findings relevant to bioactive compound production or human health.

## 3. Nutritional and Functional Composition of Microalgae

Microalgae are unicellular photosynthetic organisms with a relatively simple cellular architecture, typically consisting of a rigid cell wall (composed of cellulose, glycoproteins, or polysaccharides depending on species); a plasma membrane; chloroplasts; mitochondria; a nucleus; and lipid bodies. Valorisation strategies primarily target intracellular components such as proteins; lipids (including EPA, DHA, and GLA); pigments (e.g., chlorophylls, phycobiliproteins, and carotenoids); polysaccharides; and polyphenols. Cell wall disruption techniques (e.g., mechanical, enzymatic, or ultrasound-assisted) are commonly employed to release these compounds. Downstream valorisation involves processes such as solvent extraction, supercritical fluid extraction, or membrane separation, tailored to preserve compound bioactivity and purity. These high-value bioactives are then used in nutraceuticals, functional foods, cosmetics, and pharmaceuticals [5,8]. These attributes position microalgae as promising candidates for next-generation functional foods that support chronic disease prevention and health optimisation.

### 3.1. Major Edible Microalgae Species

The commercialisation of edible microalgae is driven by their dense nutrient profiles and health-promoting bioactive compounds. Comprehensive data on the chemical composition of major microalgae species are available in previously published reviews [28,30,31]. Global consumer interest in algae-derived health products is rising, with the market expected to exceed USD 1.5 billion by 2030 [32].

Spirulina is the most widely cultivated edible microalga, contributing over 60% of the total global microalgal biomass. It provides up to 70% protein by dry weight, contains all essential amino acids, and offers key micronutrients such as iron and phycocyanin, making it a valuable tool for combating protein–energy malnutrition [33].

*Chlorella vulgaris* is similarly rich in protein (50–60% dry weight) and chlorophyll and is among the few plant-based sources of bioavailable vitamin B12, contributing to its popularity in supplements aimed at detoxification and immune support [8].

*H. pluvialis* is best known for its astaxanthin content, the most potent naturally occurring antioxidant, reaching up to 4% dry weight. This microalga underpins a market projected to exceed USD 1.2 billion by 2023 due to its roles in anti-ageing and cardiovascular protection [5].

*Nannochloropsis* spp. are cultivated primarily for their omega-3 PUFA content, particularly EPA, providing a plant-based alternative to fish oil and supporting heart and brain health [34].

*Scenedesmus almeriensis* is another emerging species attracting industrial interest. Rich in lutein, it is cultivated mainly in closed photobioreactor systems to ensure high pigment yields. Its biomass and lutein-rich extracts are increasingly used in functional foods and supplements aimed at supporting eye health and reducing oxidative stress [35].

*T. suecica*, while less commercially prominent, is valued for its phytosterols, lutein, and antioxidant carotenoids that support cholesterol balance and ocular health [28].

### 3.2. Key Bioactive Compounds

Microalgae synthesise a diverse range of bioactive compounds that contribute significantly to their classification as functional food ingredients with therapeutic potential [36]. Among the most widely studied are PUFAs, particularly EPA and DHA, which are abundantly produced by microalgae such as *Nannochloropsis*, *Isochrysis*, and *Schizochytrium*. These long-chain omega-3 PUFAs play essential roles in cardiovascular, neurological, and inflammatory health, making microalgae a sustainable and vegan-friendly source of these vital lipids [33].

Carotenoids are another hallmark of microalgal bioactivity. *H. pluvialis* is the richest natural source of astaxanthin, a potent antioxidant carotenoid linked to skin protection, anti-ageing, and inflammation reduction. Other microalgae, including *Dunaliella salina* and *Chlorella vulgaris*, produce significant quantities of β-carotene and lutein, which contribute to visual health and lipid homeostasis [37].

Phenolic compounds, although typically present in lower concentrations than in terrestrial plants, are still significant in microalgal species such as *Tetraselmis* and *Phaeodactylum*. Commonly identified phenolics include gallic acid, ferulic acid, and catechins. These phenolics demonstrate anti-inflammatory activity, particularly through cytokine modulation involving IL-6 and TNF-α [38].

Phycobiliproteins, such as phycocyanin from Spirulina, offer water-soluble antioxidant benefits and have shown neuroprotective and immunomodulatory effects. These bioactives enhance cellular antioxidant defences and suppress inflammatory markers, making them highly relevant in nutraceutical applications [16].

Another emerging class is bioactive peptides derived from the enzymatic hydrolysis of microalgal proteins. These peptides have shown promise in preclinical studies for antihypertensive, antidiabetic, and even anticancer effects, offering new avenues for therapeutic food formulations [39].

Finally, other functional components, including phytosterols; mycosporine-like amino acids (MAAs); vitamins (B12, E, and K); and essential trace minerals such as selenium and iron, enhance the nutritional and therapeutic profiles of edible microalgae. These elements contribute synergistically to their health-promoting potential [32,40].

### 3.3. Nutritional Variability and Influence of Cultivation

The biochemical composition of microalgae is highly dynamic, shaped by environmental, genetic, and operational factors. Cultivation conditions, such as light intensity, temperature, nutrient availability, and growth phase, play a critical role in determining the levels of proteins, lipids, pigments, and bioactive compounds.

For instance, high light intensity stimulates carotenoid synthesis in species like *Dunaliella salina* and *H. pluvialis*, where β-carotene and astaxanthin serve as photoprotective agents [37]. In contrast, nutrient stress, particularly nitrogen limitation, shifts cellular metabolism toward lipid accumulation at the expense of protein production [41].

Salinity alterations can enhance the production of osmolytes like glycine, betaine, and extracellular polysaccharides, while temperature changes influence the degree of FA desaturation, affecting lipid profiles in microalgae such as *Tetraselmis* and *Nannochloropsis* [32].

The stage of growth also matters: During exponential growth, microalgae prioritise protein synthesis, while the stationary phase favours the accumulation of storage lipids and secondary metabolites like pigments [36]. Cultivation systems, whether open ponds or closed photobioreactors, further influence the biomass yield, purity, and consistency of the bioactive content.

### 3.4. Analytical Determination

The complex biochemical nature of microalgae necessitates the use of a broad spectrum of analytical methodologies for precise profiling of their nutritional and bioactive constituents. Spectrophotometry is commonly used for the rapid quantification of chlorophylls, carotenoids, and phycobiliproteins [16]. However, for more specific compound identification, high-performance liquid chromatography (HPLC), often coupled with diode-array detection (DAD) or mass spectrometry (MS), is widely used to analyse carotenoids, phenolics, and vitamins [38].

Lipid profiles are typically assessed using gas chromatography (GC) with flame ionisation detection (FID) or MS after conversion to FA methyl esters (FAMEs), providing the precise quantification of PUFAs [33].

The protein content is generally measured using the Bradford assay, while advanced peptide profiling and identification of bioactive peptides are carried out using LC-MS/MS [39].

To assess the bioaccessibility and bioavailability of these compounds, in vitro digestion simulations following protocols such as INFOGEST and intestinal cell models like Caco-2 monolayers are increasingly used [40].

Beyond these conventional methods, recent advancements in omics technologies are revolutionising microalgae characterisation. Multi-omics approaches, including genomics, transcriptomics, proteomics, metabolomics, and lipidomics, allow for an integrated and systems-level understanding of microalgal metabolism and stress responses [42]. For example, metabolomics enables the detailed mapping of metabolic fluxes under different culture conditions, while transcriptomic and proteomic profiling help identify regulatory genes and enzymes involved in bioactive compound biosynthesis [43].

The integration of omics with machine learning and bioinformatics platforms is further advancing the field of foodomics, providing deep insight into compound–function relationships and supporting the precision formulation of functional foods [44].

## 4. Health-Promoting Properties of Microalgae Bioactives

Microalgae-derived bioactive compounds have acquired significant attention for their diverse health benefits, including antioxidant, anti-inflammatory, metabolic regulatory, and gut-modulatory effects. These functional properties make microalgae promising candidates for inclusion in functional foods and nutraceuticals aimed at preventing and managing chronic diseases.

### 4.1. Antioxidant Activity and Reduction of Oxidative Stress

Oxidative stress, resulting from an imbalance between ROS production and the body’s antioxidant defences, is implicated in ageing and a wide spectrum of chronic conditions, including cardiovascular disease, neurodegeneration, cancer, and diabetes. Microalgae offer a unique arsenal of antioxidant compounds capable of mitigating this cellular damage.

Key microalgal antioxidants include carotenoids (e.g., astaxanthin, β-carotene, and lutein); phycobiliproteins (e.g., phycocyanin); phenolic compounds; vitamins C and E; and omega-3 PUFAs like EPA and DHA [45].

Among these, astaxanthin from *H. pluvialis* is particularly potent, showing antioxidant activity thousands of times stronger than vitamin C and significantly outperforming β-carotene and vitamin E in neutralising singlet oxygen and lipid radicals [46].

Phycocyanin, a pigment–protein complex found in Spirulina, is also a powerful antioxidant. It enhances the activity of endogenous enzymes like superoxide dismutase (SOD), catalase (CAT), and glutathione peroxidase (GPx), helping to reduce lipid peroxidation and support mitochondrial function [16].

Phenolic compounds from species such as *Tetraselmis* and *Phaeodactylum* contribute to redox homeostasis by scavenging free radicals and chelating pro-oxidant metal ions [38].

Moreover, omega-3 PUFAs from *Nannochloropsis* and *Isochrysis* modulate oxidative stress through the Nrf2 signalling pathway, upregulating genes associated with antioxidant defence [21].

Finally, novel evidence suggests that microalgae may protect mitochondrial function and muscle tissue from oxidative damage, offering benefits for ageing populations and individuals with metabolic or muscular disorders [47].

These multifaceted antioxidant mechanisms support the growing recognition of microalgae as valuable contributors to health maintenance and disease prevention through oxidative stress modulation.

### 4.2. Anti-Inflammatory Effects and Immune Modulation

Chronic low-grade inflammation is a central driver of non-communicable diseases such as atherosclerosis, type 2 diabetes, neurodegeneration, autoimmune conditions, cancer, and ageing [48,49]. Microalgal bioactive compounds mitigate inflammation through diverse molecular pathways, modulating cytokine production and key signalling mechanisms.

Astaxanthin, derived from *H. pluvialis*, is particularly effective in suppressing inflammatory cascades by inhibiting the NF-κB pathway; reducing the expression of pro-inflammatory cytokines (TNF-α, IL-1β, and IL-6); and downregulating COX-2 and iNOS enzymes [38]. Clinical and animal studies support its efficacy in lowering systemic inflammation and protecting against oxidative and neuroinflammatory damage [50].

Phycocyanin, a pigment–protein complex from Spirulina, exhibits significant anti-inflammatory properties. It reduces leukocyte infiltration, inhibits pro-inflammatory enzymes, and boosts IL-10 production, thereby dampening inflammation and protecting neural tissue from microglial activation [51].

Microalgal phytosterols, such as β-sitosterol and stigmasterol, sourced from *Tetraselmis* and *Isochrysis*, reduce systemic inflammation by modulating lipid mediators and suppressing pro-inflammatory gene expression [52].

Bioactive peptides from hydrolysed proteins of Spirulina and *Chlorella* exhibit ACE inhibitory and antioxidant properties, contributing to vascular relaxation and anti-inflammatory responses [53].

At the immune level, extracts from *Chlorella vulgaris* enhance innate immune defences by stimulating macrophage activity, increasing phagocytosis, and boosting mucosal immunity via IgA secretion [33]. Collectively, these immunomodulatory and anti-inflammatory effects highlight the potential of microalgae as therapeutic agents for inflammation-related chronic disease prevention.

While bioactives like astaxanthin and phycocyanin are known to modulate key signalling pathways (e.g., NF-κB and Nrf2), their molecular effects are influenced by factors such as cell type, oxidative context, and dosage. Thus, mechanistic interpretations should be approached cautiously, and no single pathway can fully explain their biological activity across all models [54].

### 4.3. Metabolic Regulation: Impacts on Lipid and Glucose Metabolism

Microalgae-derived bioactives are increasingly recognised for their ability to regulate lipid and glucose metabolism, both of which are critical factors in the prevention and management of metabolic syndrome, obesity, and type 2 diabetes [55,56].

Marine-sourced PUFAs, particularly EPA and DHA produced by *Nannochloropsis*, *Schizochytrium*, and *Isochrysis*, have been shown to promote lipid catabolism, reduce hepatic triglyceride synthesis, and enhance lipoprotein clearance. These effects are largely mediated through the activation of PPAR-α, a key transcription factor involved in FA β-oxidation and glucose homeostasis [21].

Carotenoids, such as fucoxanthin derived from *Phaeodactylum tricornutum* and *Isochrysis galbana*, stimulate thermogenesis by upregulating uncoupling protein 1 (UCP1) expression in white adipose tissue while simultaneously activating AMPK pathways to enhance glucose uptake in skeletal muscles [22]. Microalgae-derived bioactives are increasingly recognised for their ability to regulate lipid and glucose metabolism, both of which are critical factors in the prevention and management of metabolic syndrome, obesity, and type 2 diabetes [33].

Phytosterols present in microalgae competitively inhibit cholesterol absorption in the intestines, thereby reducing low-density lipoprotein (LDL) cholesterol levels and supporting cardiovascular health [17].

Simultaneously, polysaccharides extracted from *Chlorella* and *Porphyridium* species slow carbohydrate absorption, attenuating postprandial glycaemic spikes. Moreover, bioactive peptides generated through the enzymatic hydrolysis of Spirulina proteins inhibit dipeptidyl peptidase-IV (DPP-IV) activity, thereby enhancing insulin secretion [39].

Microalgae also offer cytoprotective effects for pancreatic β-cells against oxidative stress, preserving endogenous insulin production and maintaining glucose homeostasis [57]. These multifaceted mechanisms collectively reinforce the metabolic health benefits of microalgae and substantiate their potential for incorporation into functional foods targeting metabolic disorders.

### 4.4. Gut Microbiota Modulation and Digestive Health

The gut microbiota exerts profound influences on digestion, immunity, and systemic metabolism. Increasing evidence suggests that microalgae-derived bioactives beneficially modulate gut microbiota composition and function, thereby supporting overall health [58,59].

Microalgae contain various non-digestible polysaccharides and fibres with prebiotic effects, including sulphated polysaccharides (e.g., from *Porphyridium*), β-glucans (e.g., from *Chlorella*), and xylans and rhamnose-rich polysaccharides (e.g., from Spirulina). These compounds have been shown to selectively promote the growth of beneficial gut microbes such as *Lactobacillus*, *Bifidobacterium*, and *Faecalibacterium prausnitzii*, as supported by recent studies on microalgae-derived prebiotics [60,61]. For instance, studies show that supplementation with Spirulina enhances short-chain FA (SCFA) production, particularly butyrate, which fosters colonic health and reduces systemic inflammation [21].

Sulphated polysaccharides extracted from *Porphyridium* spp. reinforce the intestinal barrier by upregulating the expression of tight junction proteins, thus reducing gut permeability and preventing metabolic endotoxemia [62].

Microalgae synthesise a diverse array of polyphenolic compounds, including caffeic acid, gallic acid, ferulic acid, catechins, quercetin, and rutin. These molecules contribute significantly to the antioxidant and anti-inflammatory properties attributed to various microalgal species. In particular, polyphenols from *Tetraselmis* and *Phaeodactylum* have been shown to undergo microbial biotransformation in the gut, resulting in the production of bioactive phenolic acids. These metabolites can exert systemic anti-inflammatory effects and bolster host antioxidant defences [63].

Extracts from *Chlorella vulgaris* have been demonstrated to bolster mucosal immunity by stimulating secretory IgA production and modulating dendritic cell function [64]. Moreover, certain microalgae exhibit antimicrobial properties, aiding in the prevention of gut infections and supporting microbiota recovery following antibiotic therapy.

**Figure 2:**
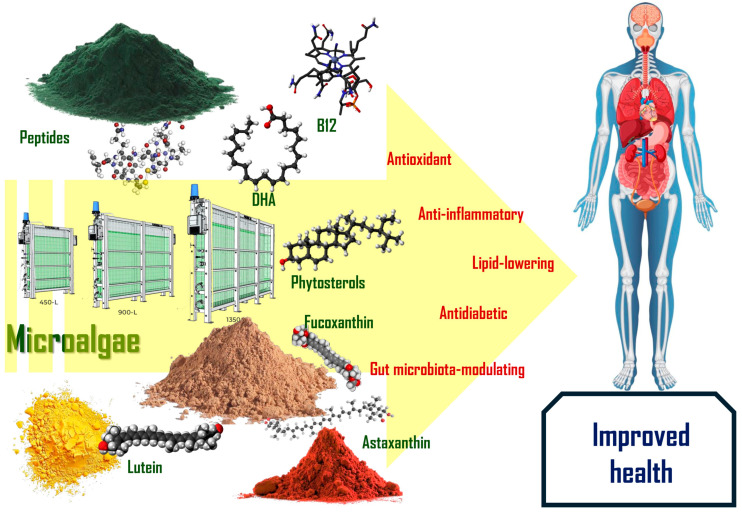
Key microalgae-derived compounds and their associated health benefits. Bioactives such as peptides, DHA, phytosterols, fucoxanthin, lutein, and astaxanthin contribute to antioxidant, anti-inflammatory, lipid-lowering, antidiabetic, and gut microbiota-modulating effects, ultimately supporting improved human health.

Altogether, microalgae not only nourish beneficial gut microbes but also reinforce intestinal barrier integrity, stimulate mucosal immunity, and produce microbial-derived metabolites that contribute to systemic health. As illustrated in Figure 2, the major bioactive compounds found in microalgae contribute to a wide range of health-promoting effects, including antioxidant, anti-inflammatory, lipid-lowering, and antidiabetic actions. These attributes position microalgae as promising dietary interventions for maintaining gut health and preventing dysbiosis-related diseases.

## 5. Evidence from Preclinical and Clinical Studies

Researchers have extensively investigated the health-promoting potential of microalgae-derived bioactive compounds through both preclinical and clinical studies. These investigations provide growing support for their use in functional foods, dietary supplements, and therapeutic applications. This section summarises evidence from in vitro systems, animal models, and human trials while identifying key gaps for future clinical translation.

### 5.1. In Vitro and Animal Model Findings

Preclinical research consistently shows that microalgal bioactives exert antioxidant, anti-inflammatory, antidiabetic, and metabolic regulatory effects, often comparable to conventional pharmaceuticals.

For instance, *C. vulgaris* extracts have been shown to protect human mesenchymal stromal cells from hydrogen peroxide-induced oxidative damage, reducing ROS and enhancing intracellular glutathione levels, all without compromising cell viability [65]. Similarly, purified fucoxanthin from *P. tricornutum* demonstrated potent antioxidant and antiproliferative effects in vitro, lowering chemiluminescence in activated neutrophils and HeLa cells while significantly increasing glutathione ratios [66].

Anti-inflammatory properties have also been strongly validated. Lipid fractions from *Nannochloropsis oceanica* and *Chlorococcum amblystomatis* markedly suppressed nitric oxide production and inflammatory gene expression (e.g., TNF-α and IL-1β) in LPS-stimulated macrophages [67]. Additionally, phycocyanin from Spirulina and peptides from *Chlorella* demonstrated antinociceptive and anti-inflammatory effects in murine models of formalin-induced pain and oedema [68].

Metabolic studies show that dietary supplementation with algal-derived EPA, DHA, and fucoxanthin lowers plasma glucose, improves insulin sensitivity, and reduces triglyceride levels in obese animal models. Fucoxanthin activates the AMPK pathway, enhancing glucose uptake in skeletal muscle tissue [22]. Microalgae are a valuable source of bioactive polar lipids, including glycolipids, phospholipids, and betaine lipids, which are often enriched with long-chain PUFAs such as EPA and DHA. These lipids are known to exert anti-inflammatory, cardioprotective, and lipid-modulating effects. Furthermore, bioactive polar lipids and peptides derived from *Isochrysis galbana* and *Tetraselmis* spp. have been shown to improve serum lipid profiles, modulate adipokine levels, and exhibit antihypertensive potential, underscoring their promise in managing cardiometabolic health [69].

Lastly, exopolysaccharides from *Porphyridium* and *Chlamydomonas* species have been found to fortify intestinal barrier function and beneficially alter gut microbiota in preclinical gastrointestinal models, underscoring their prebiotic promise [70].

### 5.2. Key Human Studies and Clinical Trial Outcomes

Although fewer in number than preclinical investigations, human studies offer compelling early evidence supporting the health benefits of microalgae supplementation, particularly in the metabolic, immune, and gastrointestinal domains.

A recent systematic review concluded that microalgae-derived supplements can enhance insulin sensitivity, modulate lipid metabolism, and lower systemic inflammation in individuals with metabolic syndrome and type 2 diabetes [71]. Interestingly, supplementation with Spirulina led to significant reductions in fasting blood glucose, HbA1c, and triglyceride levels after 8–12 weeks of use, with minimal adverse effects reported.

In hyperlipidaemic individuals, *C. vulgaris* supplementation reduced the total and LDL cholesterol while simultaneously improving the antioxidant status through the elevated activity of SOD and CAT. These effects were attributed to synergistic interactions between its carotenoids, chlorophylls, and dietary fibres [65].

Emerging findings also suggest cognitive and mood-enhancing potential. A recent clinical review highlighted that microalgal bioactives such as omega-3 PUFAs and carotenoids may exert neuroprotective and mood-regulating effects via the modulation of neuroinflammation and oxidative stress pathways, although more targeted trials are needed [72].

Regarding digestive health, Spirulina-derived oligosaccharides and polysaccharides have been shown to support beneficial gut microbiota, mainly *Bifidobacterium* and *Faecalibacterium prausnitzii*, which are associated with anti-inflammatory effects and improved metabolic outcomes [73].

Despite these promising outcomes, large-scale, randomised controlled trials remain limited. There is a clear need for rigorous human research to establish long-term safety, optimal dosage ranges, and efficacy across different populations and health conditions [74].

In addition to the previously cited systematic review, several individual clinical studies also support the metabolic benefits of microalgae. For instance, daily supplementation with 2 g of Spirulina for 12 weeks significantly improved insulin sensitivity and reduced triglycerides in overweight adults with metabolic syndrome [75]. Another randomised controlled trial involving 60 type 2 diabetic patients found that 3 g/day of *C. vulgaris* for 8 weeks led to meaningful reductions in fasting blood glucose and LDL cholesterol [76]. Furthermore, astaxanthin supplementation from *Haematococcus pluvialis* at doses of 6–12 mg/day has shown anti-inflammatory effects and improvements in lipid oxidation markers in hyperlipidaemic subjects [77]. These studies provide promising, though still limited, evidence of clinical efficacy.

### 5.3. Limitations and Gaps in the Existing Research

Despite significant advancements, several limitations continue to constrain the clinical translation and regulatory acceptance of microalgae-derived bioactives.

Firstly, the majority of the current evidence is derived from in vitro and animal model studies, which, while informative, limit direct extrapolation to human health outcomes without robust validation through large, well-designed clinical trials. Many existing human studies suffer from small sample sizes, short intervention periods, and a lack of rigorous, randomised, double-blind, placebo-controlled designs [78].

Secondly, methodological inconsistencies, including differences in extraction techniques, bioactive compound standardisation, and product formulations, complicate reproducibility across studies. Furthermore, bioavailability challenges, especially for lipophilic compounds such as carotenoids, remain significant. The influence of food matrices and interactions with the gut microbiota on nutrient absorption is still poorly characterised [79].

Thirdly, individual biological variability, encompassing differences in gut microbiota composition, genetic background, and baseline nutritional status, can markedly influence the effectiveness of microalgal interventions. Future research should incorporate stratified analyses and personalised approaches to better identify the population subgroups most likely to benefit [69].

Finally, regulatory barriers persist. While established species such as Spirulina and *Chlorella* have attained Generally Recognised As Safe (GRAS) status, introducing novel microalgae species or concentrated extracts often necessitates additional toxicological evaluation. Furthermore, the lack of universally accepted and substantiated health claims limits their clinical endorsement and broader functional food market integration [32].

Table 1 summarises the main edible microalgae species, their key bioactive compounds, documented physiological effects, and the principal studies supporting their functional applications.

## 6. Application in Functional Foods and Nutraceuticals

Microalgae-derived bioactive compounds present an exciting avenue for the development of functional foods and nutraceuticals, offering dense nutritional profiles alongside a wide spectrum of health benefits. Microalgae provide a sustainable source of high-value nutrients, including 40–60% protein by dry weight; essential omega-3 PUFAs (EPA, and DHA); vitamins (A, B12, C, D, and E); minerals (iron, calcium, and magnesium); and key bioactives such as carotenoids (β-carotene and astaxanthin), phycocyanin, and sulphated polysaccharides. These compounds deliver potent antioxidant, anti-inflammatory, immunomodulatory, and prebiotic effects, supporting the prevention and management of chronic diseases while promoting cardiovascular, cognitive, and mitochondrial health [80].

### 6.1. Integration of Microalgae into Food Systems

Microalgae are increasingly recognised as a valuable component of modern food systems due to their high nutrient richness, diverse bioactive profiles, and sustainable production characteristics. They supply high-quality proteins, lipids, carbohydrates, vitamins, minerals, and functional compounds suitable for a broad range of food and beverage applications [24,28,81,82].

Among the most prominent applications, dietary supplements dominate the market, particularly products featuring Spirulina and *Chlorella*, valued for their rich protein content and comprehensive micronutrient profiles [24,81]. These microalgae are formulated into powders, tablets, and capsules aimed at plant-based consumers seeking natural alternatives.

Beverage formulations represent a growing innovation space, where microalgae are incorporated to boost the antioxidant content without significantly altering taste profiles. Examples include fortified juices, smoothies, and functional wellness shots enriched with Spirulina extracts [82].

Snacks and convenience foods, such as protein bars, crackers, and chips, are also enhanced with microalgal ingredients, offering superior protein, fibre, and omega-3 PUFA content to meet the rising consumer demand for plant-based and functional snacking options [24].

Beyond supplements, microalgae contribute significantly to the formulation of functional foods targeted at specific health outcomes, including cardiovascular protection, cognitive support, and immune enhancement, largely driven by their rich profiles in EPA, carotenoids, phytosterols, and polysaccharides [83,84].

Nevertheless, sensory challenges related to marine flavours, dark pigmentation, and strong odours remain critical barriers to wider acceptance. Addressing these issues requires the use of flavour-masking technologies, decolourisation, and deodorisation strategies [24,26]. To mitigate the sensory challenges posed by the incorporation of microalgae, such as off-flavours, bitterness, and undesirable colour, several strategies have been developed. Microencapsulation techniques such as spray-drying, complex coacervation, and emulsification are widely used to entrap microalgal bioactives and reduce sensory impact. For instance, Spirulina microencapsulated in alginate and whey protein showed significantly improved acceptability in fortified yoghurt due to the better control of flavour and colour perception [85]. Flavour masking can also be achieved using citrus oils, herbs, or umami-rich ingredients, which help balance the marine-like or grassy notes characteristic of microalgae. Additionally, encapsulation technologies improve oxidative stability and allow the targeted release of bioactives in the gastrointestinal tract, enhancing both shelf life and palatability [86,87].

Moreover, economic viability remains a major hurdle, as the cost of large-scale microalgae cultivation and biomass processing remains relatively high. Investment in cost-effective cultivation technologies, such as photobioreactors and heterotrophic fermentation, alongside improvements in extraction efficiency, is urgently needed to make microalgae more accessible for mass market applications [26,83].

To illustrate the breadth of microalgae applications across the functional food and nutraceutical sectors, Table 2 summarises key edible species, their principal uses, and supporting references from the recent literature. It highlights the established roles of species such as Spirulina, *Chlorella*, *H. pluvialis*, and *Nannochloropsis*, as well as the emerging potential of species like *S. almeriensis*, *Aphanizomenon*, *Nostoc*, *Isochrysis galbana*, and *Schizochytrium* spp. [17,35,38,88,89].

### 6.2. Bioavailability Considerations and Enhancement Strategies

Although microalgae are rich sources of bioactive compounds, their effective utilisation in functional foods and nutraceuticals is often limited by bioavailability challenges. Several intrinsic and extrinsic factors compromise nutrient release and absorption, including the robust cell wall structures that restrict the liberation of intracellular nutrients, the chemical and physical instability of bioactives during processing and gastrointestinal digestion, and interactions with other food matrix components that hinder nutrient solubility and uptake [91,92].

To overcome these limitations and enhance the functional efficacy of microalgal bioactives, several technological strategies have been explored [93,94]. Encapsulation technologies have been widely investigated to protect sensitive compounds, such as carotenoids and omega-3 PUFAs, from oxidation and degradation. Advanced delivery systems, including nanoemulsions, liposomes, and hydrogels, have been shown to significantly improve the controlled release, intestinal stability, and absorption of these bioactives [26,95].

Cell disruption techniques, such as high-pressure homogenisation, ultrasonication, bead milling, and enzymatic hydrolysis, have proven effective in breaking down the resilient cell walls of microalgae, thus facilitating the release of intracellular nutrients like lipids, carotenoids, and proteins [27,93]. High-pressure homogenisation, in particular, is noted for enhancing nutrient bioaccessibility without the significant degradation of thermolabile compounds.

Mild thermal processing can also enhance the functional characteristics of microalgal biomass, including emulsification, gelation, and viscosity properties. When combined with mechanical disruption techniques, thermal treatments amplify the bioavailability of intracellular nutrients while maintaining the structural integrity of sensitive molecules [27].

Nanotechnology represents a cutting-edge approach for both cultivation enhancement and product formulation. The use of nanomaterials during cultivation can improve CO_2_ fixation, nutrient uptake, and light conversion efficiency, thereby increasing the intracellular accumulation of valuable compounds [96]. In addition, the encapsulation of microalgal bioactives into nanoparticles markedly improves their solubility, protects them against gastrointestinal degradation, and enhances systemic bioavailability.

Manipulating cultivation conditions provides another strategic route to maximise bioactive production. Adjustments to factors such as nutrient availability, salinity, light intensity, and CO_2_ concentration have been shown to stimulate the biosynthesis of lipids, carotenoids, and antioxidant molecules. Techniques like nitrogen starvation and phosphorus limitation are well documented for boosting both biomass and bioactive yields [81,97].

Altogether, improving the bioavailability of microalgae-derived bioactives is essential for unlocking their full therapeutic and functional food potential. The integration of encapsulation, cell disruption, mild processing, nanotechnology, and cultivation optimisation provides a comprehensive, multi-pronged strategy to overcome current bioavailability limitations and enhance the efficacy of microalgal-based food and nutraceutical products. Table 3 summarises the main strategies employed to enhance microalgae bioactive utilisation, outlining their descriptions, benefits, associated challenges, and key supporting references.

### 6.3. Synergistic Effects of Whole Biomass vs. Isolated Compounds

The growing interest in microalgae as functional food and nutraceutical ingredients stems from the distinct advantages offered by both whole microalgal biomass and isolated bioactive compounds. Understanding the complementary roles and potential synergies between these two approaches is essential for optimising health benefits, enhancing product efficacy, and advancing innovation in sustainable nutrition [99].

Whole microalgal biomass provides a naturally balanced matrix containing macronutrients, such as proteins, lipids, and carbohydrates, and micronutrients, including essential vitamins and minerals. This composition is complemented by a rich variety of bioactive compounds, such as carotenoids, phycobiliproteins, and polysaccharides [98]. The integration of multiple functional molecules within the natural matrix promotes synergistic interactions, leading to enhanced bioavailability and potentiated health benefits [100,101].

Beyond nutritional richness, using the whole biomass minimises the need for extensive processing and purification steps, resulting in lower production costs and reduced environmental impact. This feature aligns with sustainability goals, appealing particularly to environmentally conscious consumers seeking clean label, minimally processed products [90,102]. Moreover, whole biomass retains fibre and other structural components that may positively modulate gut microbiota and gastrointestinal health.

In contrast, the isolation of specific bioactive compounds enables the development of targeted functional products with standardised compositions. Concentrated forms of key compounds such as astaxanthin, EPA, or phycocyanin can achieve therapeutic dosages not easily obtainable from the whole biomass alone [103].

This approach allows for enhanced precision in dosage, quality control, and regulatory compliance, making isolated bioactives highly attractive for medical nutrition; dietary supplements; and therapeutic applications targeting antioxidant protection, cardiovascular health, cognitive function, or anti-inflammatory effects [103]. Furthermore, purified compounds often exhibit greater consistency across batches, facilitating clinical research and regulatory approvals for specific health claims.

However, isolation often involves complex extraction and purification processes that may be energy-intensive and costly and may alter the natural functionality of the compounds if not carefully controlled [104].

Emerging research suggests that combining the comprehensive nutritional benefits of whole microalgal biomass with the potency of isolated bioactives offers a promising strategy for next-generation functional foods and nutraceuticals. This integrated approach seeks to harness the synergistic interactions among natural nutrients while simultaneously enhancing specific physiological responses through targeted supplementation [103].

Such a strategy addresses the limitations inherent in each approach alone: While whole biomass offers holistic health support and sustainability advantages, isolated compounds allow for the achievement of clinically effective doses and more precise health interventions. Integrated formulations could enhance nutrient absorption, reduce variability in biological responses, and provide broader spectrum health protection. This trend is expected to dominate future innovations in microalgae-based functional nutrition, catering to the increasing demand for personalised and sustainable health solutions [4].

The optimal use of microalgae in functional foods and nutraceuticals will likely depend on a strategic combination of whole biomass and isolated bioactives, tailored according to specific health objectives, consumer needs, regulatory frameworks, and environmental sustainability goals. By leveraging the strengths of both approaches, it is possible to maximise the nutritional, therapeutic, and commercial potential of microalgae within evolving food systems.

## 7. Sustainability and Innovation Potential

Microalgae offer a sustainable and innovative platform for food production, providing high-quality proteins, essential FAs, vitamins, minerals, and bioactive compounds with health-promoting effects [105]. Their cultivation requires minimal land and freshwater, thrives on saline and wastewater sources, and captures atmospheric CO_2_, contributing to environmental sustainability [106,107,108]. While advances in genetic engineering and processing technologies are expanding their applications [109], barriers such as high costs and consumer acceptance challenges remain [4].

### 7.1. Environmental Advantages of Microalgae Cultivation

Microalgae have emerged as a uniquely versatile and productive biological resource, offering numerous environmental advantages that make them highly attractive for sustainable bioenergy, food, and bioproduct applications [109,110].

One of the most compelling advantages of microalgae is their high biomass productivity. Unlike terrestrial crops, which are constrained by seasonal growth cycles, microalgae are capable of continuous solar energy conversion, achieving high biomass yields per unit area [111,112]. This high productivity supports both renewable energy generation and the sustainable production of functional biomolecules.

Additionally, microalgae cultivation requires minimal arable land, as it can be performed on non-productive or marginal terrains, thus avoiding competition with food crops for fertile soils [106]. This attribute enhances food security and promotes sustainable land management practices.

Microalgae also possess a remarkable adaptability to diverse water resources. They can thrive in saline, brackish, and even wastewater environments, significantly reducing dependency on freshwater, a critical advantage in regions grappling with water scarcity [113]. Moreover, their capacity to remove excess nitrogen, phosphorus, and other contaminants positions microalgae as an effective tool for wastewater bioremediation, contributing to improved water quality and closed-loop nutrient recovery systems.

Crucially, microalgae play an important role in carbon sequestration. Through photosynthetic activity, they capture and fix atmospheric CO_2_, offering a viable strategy for industrial carbon emission reduction and supporting broader climate change mitigation efforts [114].

Microalgae cultivation is further distinguished by its capacity for year-round biomass production. Unlike traditional agriculture, which is often limited by growing seasons, microalgae can be continuously cultivated and harvested, ensuring a stable and scalable supply of biomass [115]. Furthermore, microalgae require significantly fewer chemical inputs, such as pesticides and synthetic fertilisers, thus minimising the risks of soil degradation and water pollution [116]. Although closed photobioreactor systems offer advantages such as reduced contamination risk, better control over growth conditions, and higher biomass productivity, they also incur significantly greater energy requirements for lighting, temperature control, and pumping. This results in a trade-off between contamination control and environmental impact. Life cycle assessments have shown that the energy demand for closed systems can outweigh their environmental benefits unless integrated with renewable energy sources or waste heat recovery systems [117,118].

Finally, the integration of microalgae cultivation with other industrial processes, such as biogas production and biorefinery systems, holds the potential to further enhance resource efficiency and environmental performance [119].

**Figure 3:**
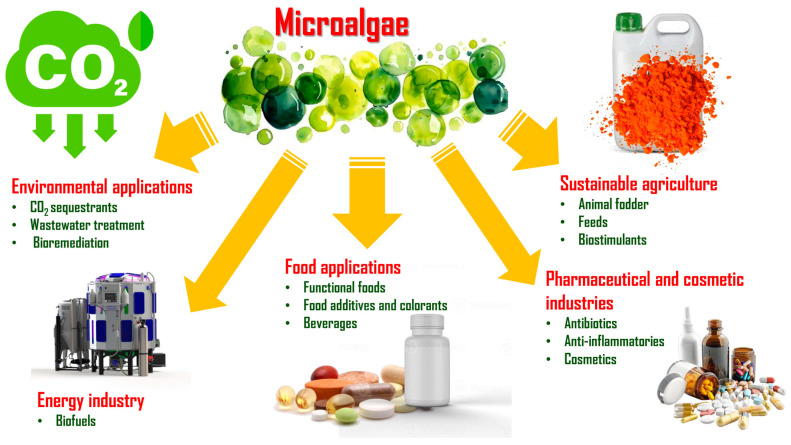
Overview of key application domains for microalgae, including functional foods, pharmaceuticals, cosmetics, agriculture, energy, and environmental management.

In conclusion, the cultivation of microalgae offers a powerful suite of environmental advantages: high productivity, flexible land and water use, wastewater treatment potential, and carbon sequestration. These characteristics firmly position microalgae as a cornerstone of future strategies for sustainable bioresource production, supporting efforts to build resilient, low-impact food and energy systems. As summarised in Figure 3, microalgae offer versatile applications across a range of industries, including functional foods, pharmaceuticals, cosmetics, sustainable agriculture, energy production, and environmental management, underscoring their value as a multifaceted bioresource.

### 7.2. Role in Future Food Systems and Dietary Recommendations

Microalgae are increasingly recognised as promising contributors to future food systems due to their exceptional nutrient density, environmental sustainability, and versatility in food applications. Their adoption aligns with the global need for resilient, low-impact, and nutrient-rich food sources that can support both health and planetary goals [5,108,120].

Key nutritional advantages include their high protein content, with species like Spirulina and *Chlorella* offering up to 70% protein by dry weight and a complete amino acid profile, alongside their richness in essential fatty acids (notably omega-3 and omega-6 PUFAs); a wide spectrum of vitamins (A, B complex, C, E, and K); minerals (e.g., iron, magnesium, and zinc); and antioxidant compounds such as carotenoids and phycobiliproteins [56,90,121,122,123]. These attributes support a range of health benefits, including improved cardiovascular, cognitive, and immune function.

From a sustainability perspective, microalgae cultivation presents notable advantages. They can be grown on non-arable land and utilise non-potable or saline water, thereby avoiding competition with traditional crops for critical resources [121,124]. Their rapid biomass productivity and year-round harvest potential further strengthen their role in sustainable agricultural practices. In addition, their carbon sequestration capability through photosynthesis positions microalgae as contributors to climate change mitigation strategies.

However, the integration of microalgae into mainstream dietary recommendations faces several challenges. One of the foremost barriers is consumer acceptance. The sensory characteristics of microalgal biomass, such as colour, taste, and odour, can be perceived as undesirable, limiting their use in conventional foods [56]. Significant research efforts are underway to improve the sensory profiles of microalgal ingredients through processing technologies, strain selection, and formulation strategies.

Technological hurdles also present obstacles to large-scale commercialisation. Efficient cultivation, cost-effective harvesting, and the preservation of nutritional integrity during processing remain major technical challenges [104,121]. Furthermore, regulatory uncertainties concerning the classification, safety evaluation, and approval of novel microalgae-based ingredients also impede market expansion [29,56].

Despite these challenges, regulatory agencies around the world have increasingly recognised microalgae as safe and viable food ingredients. In the United States, several species, such as Spirulina and *Chlorella*, have been granted GRAS (Generally Recognised As Safe) status by the FDA, allowing their use in a wide range of food products. In the European Union, the European Food Safety Authority (EFSA) has approved certain strains like *Haematococcus pluvialis* and *Tetraselmis chuii* as novel foods under Regulation (EU) 2015/2283. Asian markets, particularly Japan and South Korea, have also incorporated microalgae into their functional food frameworks through positive list systems or food with health claims (FHC) approvals. Despite these advances, integration into national dietary guidelines remains limited, highlighting the need for stronger alignment between emerging science and public nutrition policies.

## 8. Conclusions and Future Directions

Microalgae stand at the forefront of innovation in sustainable nutrition, offering a diverse and potent range of bioactive compounds with proven health-promoting properties. As highlighted in this review, species such as Spirulina, *Chlorella*, *Haematococcus*, and *Nannochloropsis* are exceptional sources of high-quality proteins; omega-3 PUFAs; essential vitamins; minerals; and unique phytochemicals like astaxanthin, phycocyanin, and sulphated polysaccharides. These bioactives exert significant antioxidant, anti-inflammatory, immunomodulatory, and metabolic regulatory effects, contributing to the prevention and management of chronic diseases.

The incorporation of microalgae into functional foods and nutraceuticals is expanding rapidly, propelled by advances in cultivation technologies, bioavailability enhancement strategies, and a growing consumer demand for sustainable and plant-based nutrition. Encapsulation, cell wall disruption, and nutrient manipulation have improved the stability, bioactivity, and sensory properties of microalgae-derived ingredients, facilitating their integration into a wide range of food matrices.

Moreover, the environmental advantages of microalgae cultivation, minimal land and freshwater requirements, carbon sequestration capabilities, and potential for wastewater bioremediation position these organisms as vital components of future resilient food systems. Their ability to align nutritional excellence with ecological sustainability addresses two of the most urgent global challenges: improving public health and reducing the environmental footprint of food production.

Nonetheless, several hurdles remain to be overcome. Clinical evidence supporting the health benefits of microalgae is still limited compared to preclinical findings, necessitating more large-scale, long-term human studies to substantiate efficacy claims. The standardisation of cultivation methods, extraction processes, and bioactive quantification is crucial to ensure consistency, reproducibility, and regulatory acceptance. Furthermore, consumer acceptance challenges, including sensory attributes and cost, must be addressed through targeted product development and education.

Looking ahead, the strategic integration of microalgae into future dietary recommendations, coupled with innovations in biotechnology and food science, holds the promise of transforming global nutrition. Personalised nutrition approaches leveraging microalgal bioactives tailored to individual health needs may become a hallmark of next-generation functional foods. Through continued interdisciplinary research, investment, and regulatory harmonisation, microalgae have the potential to transition from niche applications to the foundational elements of sustainable, health-promoting food systems worldwide.

## Figures and Tables

**Table 1:** Main bioactive compounds, physiological effects, and scientific evidence of the edible major microalgae listed in alphabetical order. Abbreviation: GLA, gamma-linolenic acid.

Microalgae Species	Main Bioactive Compounds	Source Type (Extract/Isolated)	Primary Physiological Actions	Evidence (Preclinical/Clinical)	References
*Chlorella vulgaris*	Proteins, chlorophyll, B12, carotenoids, polysaccharides	Whole biomass	General health benefits, detoxifying, gut health	↓ LDL, ↑ HDL, antioxidant improvement (clinical)	[11,65]
		Extract	Antioxidant, hepatoprotective	↑ GSH, ↓ ROS (in vitro)	[38]
		Polysaccharides (isolated)	Immunomodulation	Cytokine regulation (preclinical)	[65]
		Peptides (isolated)	ACE inhibition, antioxidant	↓ BP markers (in vivo)	[39]
		Lipid extract	Anti-inflammatory (skin)	↓ IL-6 expression in NHDF cells	[28]
*Dunaliella salina*	β-carotene (up to 10%), lutein	Whole biomass/lipid extract	Antioxidant, skin and eye health	ROS scavenging, lipid membrane protection (preclinical)	[37]
*Haematococcus pluvialis*	Astaxanthin, carotenoids, PUFAs	Astaxanthin (isolated)	Antioxidant, anti-inflammatory, photoprotection	↓ IL-6, TNF-α, NF-κB; ↑ mitochondrial function	[16]
*Isochrysis galbana*	DHA, fucoxanthin, phytosterols, proteins	Whole biomas/lipid extract	Antioxidant, lipid-lowering, thermogenic, neuroprotective	AMPK activation, PPAR modulation, UCP1 upregulation	[22]
*Nannochloropsis* spp.	EPA, chlorophyll, carotenoids, phytosterols	Whole biomass	General lipid support	↑ lipid profile (animal)	[33]
		Lipid extracts	Anti-inflammatory	↓ cytokines (in vitro)	[67]
		EPA (isolated)	Cardioprotective, lipid-lowering	Improved lipids (preclinical)	[33]
		Phytosterols (isolated)	Anti-inflammatory	↓ inflammatory cytokines	[67]
		Lipid extract	Antioxidant, anti-inflammatory (skin)	↓ ROS, ↓ IL-6 (in vitro)	[67]
*Phaeodactylum tricornutum*	Fucoxanthin, EPA, phenolics, fibres	Extract	Antioxidant	↑ antioxidant capacity (in vitro)	[38]
		Fucoxanthin (isolated)	Thermogenic, hypoglycemic	↑ AMPK, ↓ glucose and weight (in vivo)	[22]
		Phenolic acids (isolated)	Antioxidant	Protective in gut and metabolic models	[38]
*Scenedesmus almeriensis*	Lutein, carotenoids, proteins	Whole biomass/lutein-enriched extract	Antioxidant, eye health protection, anti-inflammatory	ROS scavenging, lipid membrane protection (preclinical)	[35]
*Schizochytrium* spp.	DHA, EPA	Whole biomass	Cardiovascular health, anti-inflammatory	PPAR-α activation, lipid metabolism modulation (preclinical)	[21,33]
Spirulina (*Limnospira platensis*)	Phycocyanin, GLA, proteins, polysaccharides, B vitamins	Whole biomass	Antioxidant, metabolic regulation, gut health	↓ HbA1c, improved lipids (clinical)	[12,21]
		Extract	Anti-inflammatory	↓ IL-6, ↑ SOD/CAT (in vivo)	[12,21]
		Phycocyanin (isolated)	Antioxidant, anti-inflammatory	↓ oxidative stress markers	[21]
		Peptides (isolated)	ACE inhibition	↓ BP, improved vascular function	[39]
		Lipid extract	Anti-inflammatory (skin)	↓ IL-6 in fibroblast models	[12]
*Tetraselmis suecica*	Lutein, phytosterols, proteins, phenolics	Whole biomass	General cardiovascular support	↓ cholesterol (in vivo), cytokine modulation	[17]
		Extract	Antioxidant, cholesterol-lowering	Improved lipid and antioxidant profiles	[38]
		Phytosterols (isolated)	Hypocholesterolemic	↓ LDL, ↑ bile acid excretion	[17]
		Lutein/Phenolics (isolated)	Antioxidant, antimicrobial	↑ antioxidant enzymes, ↓ pathogens	[38]

**Table 2:** Overview of the major edible microalgae species and their applications in functional foods and nutraceuticals, organised by alphabetical order.

Microalgae Species	Applications	Market Status	References
*Aphanizomenon*	Supplements, functional foods (traditional health applications)	Commercial	[88]
*Chlorella vulgaris*	Supplements, functional foods, beverages, snacks (rich in proteins, chlorophyll, and fibres)	Commercial	[24,28,82]
*Dunaliella salina*	Supplements, functional foods (rich in β-carotene for antioxidant support)	Commercial	[28,35]
*Haematococcus pluvialis*	Supplements, functional foods (rich in astaxanthin for antioxidant and anti-inflammatory support)	Commercial	[28,35]
*Isochrysis galbana*	Supplements, functional foods (rich in DHA, EPA, and fucoxanthin for cardiovascular and metabolic health)	Under development	[26,90]
*Nannochloropsis gaditana*	Supplements, functional foods (rich in omega-3 PUFA, especially EPA)	Commercial	[35]
*Nostoc*	Supplements, functional foods (traditional health applications)	Under development	[89]
*Phaeodactylum tricornutum*	Supplements, functional foods (rich in fucoxanthin and EPA for anti-obesity and antioxidant effects)	Under development	[21,22]
*Porphyridium* spp.	Supplements, functional foods (rich in sulphated polysaccharides for gut health and prebiotic effects)	Under development	[62,70]
*Scenedesmus almeriensis*	Supplements, functional foods (rich in lutein for eye health and antioxidant support)	Under development	[35]
*Schizochytrium* spp.	Supplements, functional foods (rich in DHA for cognitive and cardiovascular health)	Commercial	[26,90]
Spirulina (*Limnospira platensis*)	Supplements, functional foods, protein powders, and snacks (rich in proteins, phycocyanin, and GLA)	Commercial	[24,28,82]
*Tetraselmis suecica*	Supplements, functional foods (rich in phytosterols and polyphenols for heart health)	Under development	[17]

**Table 3:** Technological approaches for optimising the utilisation of microalgae bioactives, listed by alphabetical order.

Strategy	Description	Benefits	Challenges	References
Cell Disruption	Breaking robust cell walls using high-pressure homogenisation or ultrasonication	• Increases lipid and protein bioaccessibility • Boosts nutrient digestibility by ~2–3x	High energy consumption and scalability issues	[27,93]
Encapsulation Technology	Wrapping bioactives (e.g., astaxanthin) in protective layers like liposomes or nanoemulsions	• Improves compound stability during storage • Enhances solubility and intestinal absorption	Costly production and potential regulatory hurdles	[26,95]
Nanotechnology	Using nanoparticles to improve light capture and nutrient delivery in cultivation (e.g., enhancing DHA production)	• Improves microalgae growth rates • Protects sensitive bioactives from degradation	Safety concerns regarding nanoparticle ingestion	[96]
Nutrient Manipulation	Adjusting nutrients (e.g., nitrogen starvation) or light to stimulate bioactive production	• Boosts the accumulation of target compounds like carotenoids or PUFAs • Enhances antioxidant content	May reduce overall biomass productivity	[97,98]
Thermal Processing	Applying mild heat to facilitate the extraction of proteins, lipids, and pigments	• Improves rheology (gel strength, viscosity) • Increases bioactive release	Potential degradation of heat-sensitive bioactives	[27]

## Data Availability

No new data were created or analysed in this study. Data sharing is not applicable to this article.
